# Economic and non-economic determinants of Iranian pharmaceutical companies’ financial performance: an empirical study

**DOI:** 10.1186/s12913-019-4735-4

**Published:** 2019-12-30

**Authors:** Mohammadreza Masoumi, Farbod Ebadi Fard Azar, Aziz RezaPour, Mohsen Mehrara

**Affiliations:** 1 Department of Health Economics, School of Health Management and Information Sciences, Iran University of Medical Sciences, Tehran, Iran; 2 Health Management and Economics Research Center, Iran University of Medical Sciences, Tehran, Iran; 3 Department of Economic, University of Tehran, Tehran, Iran

**Keywords:** Pharmaceutical industry, Macroeconomic factors, Health economics, Iran

## Abstract

**Background:**

The pharmaceutical industry in Iran is influenced by various parameters such as internal factors caused by the financial information of each economic unit and external factors including major economic and non-economic variables.

**Methods:**

This study is aiming to examine the effect of such variables on the stock return of 34 pharmaceutical companies in the Tehran Securities Exchange market using quarterly data from 1995 to 2016. In this research, an autoregressive model was utilized to examine the way that variables affect the stock market index. In such patterns, there is no need for explicit short-term structural relationships and structural knowledge is extracted from causal relationships. Finally, to analyze the results, impulse-response functions, forecast error variance, and historical decomposition were collected.

**Results:**

Results of this research show that positive shock to the variables, namely the currency rate, collection period of quests, and healthcare costs lead to a decrease in the return of pharmaceutical companies. On the other hand, a positive shock to the variables such as GDP, and money volume, leads to an increase in the stock return of pharmaceutical companies.

**Conclusion:**

Different factors contribute to the stock return of pharmaceutical companies. Among the variables examined in this study, market currency rate, money volume, pharmaceutical sector inflation, bank interest rate, GDP in the healthcare sector, healthcare costs, and collection period of quests have the most effect on describing changes within the stock return of pharmaceutical companies.

## Background

Medicine is one of the most important products in today’s commerce in terms of influence on public healthcare. Nowadays, the pharmaceutical industry is considered as a key and substantial industry in the world. Such an industry guarantees one of the most important factors of development. The volume associated with the global pharmaceutical market will grow up from 887 billion dollars in 2010 to about 1400 billion dollars in 2020, which indicates a 58% growth in 10 years [1].

Iran has a prominent background in medical practice in the world. For instance, in the Mesopotamian era, physicians used to carve diagnoses and prescriptions into stone tablets. The current Iranian pharmaceutical industry initiated its operation a century ago in Tehran with the opening of the first modern-style drugstore by German, French, and Austrian pharmacists. Pharmaceutical training was commenced by European experts at Darolfonoon, which played a very important role in the Iranian pharmaceutical industry [2]. After the 1979 Iranian Revolution, two major events caused fundamental changes: 1) nationalization of the pharmaceutical industries and 2) the generic scheme. The privatization of public-owned companies and the transition to the semi-governmental sector was a major step taken by the government during 1988–1993. Most entrepots of the pharmaceutical industry were either relying on the former regime or foreigners who left the country after 1978. Thus, the pharmaceutical system of the country began its operation as a new sector. In the 1980s, the Iran-Iraq War was the main driver of the industry especially for the strict monitoring of the market [3]. Afterward, the pharmaceutical industry in Iran became one of the key industries to boost economic growth. The total value of the industry was 4 billion dollars in 2011, experiencing an average growth rate of 15% during 2007–2011.

In 2014, the domestic pharmaceutical market was estimated 2.35 billion USD and it is anticipated to rise to 3.31 billion USD in 2019, with a compound annual growth rate (CAGR) of 7.5%. In terms of medicine and medicaments, there are about 56 pharmaceutical companies in Iran, of which 37 are stock-traded, representing more than 90% of the total products. Moreover, there are 123 registered importers, 30 specialized distributors, and 10,000 drugstores. In the case of total domestic supply, 96% of the medicine is locally produced and only 4% belong to import. However, in terms of value, 55% of the market belongs to local producers and 45% to imports [2].

Although total production by quantity has increased about 1.5 times over the past decade, the value of production has increased by about 12 times. The main reason for this jump can be due to the devaluation of local currency and the high dependency of production on imported raw materials, which led to an increase in the price of medicines. On the other hand, the embargoes on the Iranian economy restrict the supply of raw materials. In short, due to the sanctions imposed on the local banks, doing overseas business was not possible through the letter of credits to supply raw materials and these companies had to pay cash instead. Also, a volatile exchange rate significantly raised the financial costs of these companies. Furthermore, the decline in government revenues followed by sanctions has led to delayed payments to hospitals or public pharmacies to pharmaceutical companies, which finally increased paying periods and financial costs.

The pharmaceutical industry faces numerous macroeconomic challenges including an increased cost of health care, pricing policy, R&D, pharmaceutical innovation, economic uncertainty, political and economic shocks, structural changes, new demands from patients, amending the regulation, and competition in the markets [4].

The Tehran Stock Exchange (TSE) as the largest stock exchange of Iran was first established in 1967. At the outset, only six companies were listed in TSE. The history of the Iranian stock exchange can be divided into four periods. In the first period, which dates to the pre-Islamic Revolution of Iran, by the year 1978, several companies admitted to the stock exchange such that the number of its members reached 105. With the victory of the Islamic Revolution in Iran, the TSE entered a new stage. Political events and the onset of a war at that time, as well as subsequent economic events, including the merger of banks and insurance companies, reduced the number of companies listed on the stock exchange from 105 companies in 1978 to 56 at the end of the year 1989. The third period began in the year 1989 and was accompanied by the formation of government policies to expand the capital market. The number of companies admitted to the stock exchange reached about 250 in the year 1996. The start of the fourth-time period on the Iranian stock market coincided with the boom of this market in the year 2003. One of the most important developments in the history of stock markets in Iran was the adoption of a new law, i.e., the Securities Market Law, in December 2009. This law remedied some of the shortcomings and deficiencies in the primary law, by which it allowed for extensive stock exchange development in Iran. The launch of the online trading system in the year 2011 was another turning point in expanding the trading of the Iranian stock market, both in terms of the value of the transactions and the inclusiveness of the community.

Over the past 10 years, Tehran’s stock market has seen two unprecedented increases. The first increase comes in 2012, after a three-fold rise in the dollar price, by which the TSE also experienced more than 3-fold growth and declined thereafter. The second unprecedented increase in the stock market is in 2018 after the sharpening of the international sanctions and the increase in the dollar prices, the TSE index has experienced more than 3-fold growth in the past 1 year and has reached 330,000 in Oct 2019. In 2019, the main TSE index has grown more than 78,000 points since the beginning of the fiscal year (March 21) to August 11.

The number of listed companies increased to 530 by early 2019. The Pharmaceutical industry is one of 37 active sectors in the Tehran Securities Stock Market. This profitable sector has the least possible risks associated with comparison to other sectors such that its average return was 160% in 2013 [5]. Examining the activity status of a company or an industry in the stock market can clarify the level of its efficiency to some degree by evaluating the level of success and performance quality in a competitive environment. Therefore, examining the stock return of the company or industry indicates the performance and reflects the factors affecting on it [6].

Based on works published by Markowitz, Sharpe (1964) and Lintner (1965), perceived total market risk can be divided into two major groups of systemic and non-systemic. The first group, i.e., systemic or inevitable risk, is not devoted to one or some enterprises but relates to the whole market. Among the factors influencing this risk, we can mention elements such as the major policies of the administration. Systemic risk, which is created due to general movements of the market, simultaneously affects the total price of securities present in the financial market and cannot be removed by the variety in an investment portfolio. The factors contributing to provide this type of risk include economic, social, and political developments such as currency rate changes, commercial cycles, monetary, and financial policies of the state, inflation, etc. Because this risk is associated with the total status of the market and its fluctuations, it cannot be reduced by a security basket that is varied proportionally. The risk also is called non-deductible or inevitable. In the second group, the non-systemic risk stems from specific characteristics of the company such as the type of product, capital structure, and major stockholders. This type of risk is only dedicated to the same asset and if the asset portfolio is created, they annul each other and disappear [7, 8]. P/E ratio, asset return, the return of stockholder rights, profit/sale ratio, and collection period of the quests are among internal variables affecting the stock return. Among these factors, the collection period of quests is the most significant factor.

In recent years, there have been numerous studies on the variables affecting the stock return of the securities market. Studies provided in this area can be generally divided into two groups. The first group focuses on examining the major economic variables’ effects on the stock index of the companies. Heidari et al. (2019), Peiro (2016), Pardhan (2015), and Chen (2007) are in this category [9–12]. The second group is dedicated to examining the effect of monetary and financial policies on the stock index of pharmaceutical companies. Agnello et al. (2011) and Laopodis (2009) are included in this group [13, 14].

According to previous studies [9–14], there are some economic factors that may contribute to the performance of pharmaceutical companies in the stock market. Due to over-importing activities from pharmaceutical companies, currency rate fluctuation risk has a significant influence on these companies because over 50% of raw materials for pharmaceutical companies are imported from other countries. In terms of the financial market, the low-interest rate makes an investment in the securities market an interesting choice [15]. Economic development is another effective factor in this regard, which is associated directly with the stock price according to Gordon’s equation [16]. Based on the asset portfolio theory, an increase in money volume leads to increasing demands for the stock and accordingly increasing its price [17]. The effect of inflation on stock price is not also clearly explicit such that different theories have been proposed in this regard. However, generally, an incremental trend of inflation will act as a barrier against economic development in the long-term and will have a negative effect on stock price [18].

Moreover, there are some non-economic factors such as political factors, technological factors, social factors, rules, and regulations that control the securities market.

The relationship between macroeconomic factors and pharmaceutical industry return or its volatility has not been studied in most countries, especially in Iran. Dierks et al. (2016) investigated “macro-economic factors influencing the architectural business model shift in the pharmaceutical industry” to understand the macroeconomic factors responsible for the business model revolution in order to obtain a competitive advantage over the market players [19]. In another work, Pardhan et al. (2015) explored the relations between economic growth, oil price, stock market size, and three other main macroeconomic indicators for the G-20 Countries during the period 1961–2012. Their results in both the long-run and short-run show that real economic growth responds to different stock market depth steps [11]. Gonzalez and Gimeno (2008) analyzed the impact of fiscal policy on stock volatility of the pharmaceutical companies in the New York Stock Exchange by tacking 20 stocks regularly for 5 years. A Markov Regime Switching model was used to investigate the stock return volatility. They found two low and high volatility states that showed Financial Analysts increases the probability of being in a state of high volatility [20]. Several studies have investigated the Iranian pharmaceutical market. The study of Heidari et al. (2019) is the most relevant one to the subject of this paper. By assessing impulse-response and decomposing variability, the findings of their study show that during the study period, money growth and health care inflation are the most important factors in Iran’s pharmaceutical industry [9]. Chizari et al. (2016) examined the effect of intellectual capital on the performance of pharmaceutical companies listed in TSE. The results show that the value-added coefficient of intellectual capital has a major impact on market performance, and among its components employed or physical capital has the greatest impact on market performance variables [21]. Mohammadzadeh et al. (2013) examined the relationship between the profitability of pharmaceutical companies and the capital structure from 2001 to 2010. As a result, a significant negative correlation exists between profitability and capital structure [22]. Zartab et al. (2013) investigated the relationship between stock return and fundamentals using the panel data from 22 pharmaceutical companies in TSE. The results show that variation in the pharmaceutical company’s stock return can be understood by taking into account the, debt-equity ratio, working capital to total asset, current ratio, net profit margin, operating cycle, market share, the inflation rate of medicinal products prices, total asset, and exchange rate [23]. Rasekh et al. (2012) investigated the R&D activities of 11 pharmaceutical companies and the critical factors affecting these activities. They concluded that some company internal factors such as management commitment, human resource management, information technology, and financial management [24] must be considered in this regard.

This study is different in some aspects than the previous literature. First, the pharmaceutical industry and influential variables were selected specifically for the industry. For example, the Gross Domestic product rate for the healthcare and medicine sector, inflation in the pharmaceutical sector, healthcare costs, and the collection period of quests for the pharmaceutical companies are specifically selected as influencing factors in the pharmaceutical industry. Accordingly, by choosing a group of stock market companies in comparison to all stock market companies, the study becomes more robust. The second fact is that this study has taken non-economic factors as well as financial information into consideration other than effective economic factors. The level of contribution for all such variables is so high and, to some extent, unknown [25]. Finally, this study has taken fundamental characteristics of the Iranian economy into consideration via identification assumptions. Characteristics such as fiscally-dominated monetary policy or government exogenous behaviors are essential to analyze an economy like the Iranian one.

We employ a Structural Vector Autoregressive model (SVAR) as the main toolbox of macroeconomic policy analysis to answer the research question. SVAR models are often used by researchers to study the effects that fundamental economic shocks may have on macroeconomic variables. Notably, SVAR models need certain assumptions to identify the structural shock of interest from the time-series properties of reduced-form VAR models. The advantage of this approach is that the researcher can analyze shock effects without imposing a full-edged general equilibrium model to interpret the dynamics of the data. Accordingly, first, a set of related variables was chosen to form a macro-econometric model. Next, an identification approach was introduced to fulfill the fundamental characteristics of the Iranian economy and, finally, the analytical tools were used to extract the results for policy recommendation.

## Methods

### Materials

To examine the relationship between major economic or non-economic factors and stock return of pharmaceutical companies, all listed companies of the TSE market between 1995 to 2016 were considered. Next, financial information for 34 active pharmaceutical companies is extracted. Moreover, major economic data are extracted from the Central Bank of Iran and the Statistical Center of Iran.

### Data analysis

In order to examine the multi-variable behavior of time series, it is necessary to consider mutual relationships between these variables in the form of a concurrent equation system. As Sims (1980) indicates, if there is a real concurrency between a set of pattern variables, we must consider all variables as the same and there is no room for pre-judgment about which variables are endogenous or exogenous. In this way, he provided his model, i.e., VAR [26].

VAR was introduced by Sims (1972) as a replacement for macro-measurement patterns [27–29]. VAR patterns are based on experimental implicit relationships among data and concurrent. In this model, the system of equations is considered in a reduced form, in which any of the endogenous variables regress on their lags and other variable lags. Therefore, in such patterns, there is no need to indicate short-term structural relationships with structural science coming from causal relationships between pattern variables. In particular, when there is no detailed information about how real-world process or determinative elements of variables are, it is inevitable to appeal to VAP patterns. In this approach, previous theories and knowledge of the researcher are only used to determine variables that should be associated with the pattern.

In its general form, it is possible to show VAR pattern with n endogenous variables and p lags, with matrix indexing as follows:

1 $$ {Z}_t=C+\sum \limits_{i=1}^p{B}_i{Z}_{t-i}+{e}_t $$

where Z_t-i_ is valued with lag in variables, e_t_ is the vector of n*1 statements of interference, Z_t_ is the vector of n*1 variables of the model, and B_i_ is a K*K matrix of the constant-coefficient.

### The process of estimating the VAR model

Affirmation: 1) Determining endogenous variables according to economic theories, experimental evidence, and experiences; choosing variables that are about to be entered in the VAR model is provided according to common economic theories, 2) Converting time series into logarithmic ones, etc., and 3) Providing quarter mediators and algebraic statements.

Determining optimum lag: VAR degree plays an important role in analyses related to this model. Akaic (AIC), Schwartz (SBC), and Hanan-Queen (HQ) measures and Likelihood Ratio (LR) are provided to determine the optimal lag length. If the lag is too short, the model will be associated with an error in affirmation, and if it is too long, the degree of freedom will increase. The least values of these measures (i.e., p) will be the determinant value to choose the order of VAR pattern.

The number of observations: While using the VAR method, the number of observations must be multiple (particularly when the number of endogenous variables increases).

Unit root test (stationarity evaluation): Accumulated VAR models are based on the premise that endogenous variables are summed in the order of one. Therefore, before implementing this method, this test seems necessary. A time-series variable is static when the average, variance, and auto-regressive coefficients remain constant with the passage of time.

### Introduction of the variables and affirmation of the model

The variables used in this study are divided into three groups. The first group is related to the information of pharmaceutical companies including 1) pharmaceutical stock index changes (TEPs), which indicate the average return of the pharmaceutical companies in the stock market, and 2) the collection period of quests for such companies (VOSOL).

Variables in the second group include major economic factors including free-market currency rate (EX), Gross Domestic product rate for healthcare and medicine sector (GDPH), inflation in the pharmaceutical sector (INFD), money volume (M1), interest rate (R), and healthcare costs (HE). Also, dummy variables, which fall in the third group, including parliament election (MAJ_EL), presidential election (PR_EL), and health transformation program (HTP) are used to capture the effect of non-economic factors on the pharmaceutical stock market index.

### Identification

Since the VAR model is based on non-orthogonal residuals, it is necessary to build a model consist of structural shocks and thereby extract the orthogonal impulse-response functions in a Structural VAR (SVAR) framework. To accomplish this, an identification strategy is required. The basic SVAR model can be shown as equation (2), where *ε* represents the structural shocks. Assuming the reduced form of VAR model as equation (3), the structural shocks can be identified through the matrix A. Note that in equation (4), Z shows the vector of endogenous variables.

2 $$ A{Z}_t=C{Z}_{t-1}+{\varepsilon}_t $$

3 $$ {Z}_t=B{Z}_{t-1}+{e}_t\;\mathrm{in}\kern0.17em \mathrm{which}\;B={A}^{-1}C\; and\;{e}_t={A}^{-1}{\varepsilon}_t $$

4 $$ {Z}_t=\left[ TE{P}_t, GDP{H}_t,E{X}_t, INF{D}_t,M{1}_t,{R}_t,H{E}_t, VOSO{L}_t\right] $$

To solve the identification problem, a minimum of n*(n-1) restrictions is needed to draw out the structural shocks. A prevalent solution is a Recursive or Cholesky identification that assumes A is a lower triangular matrix. In Cholesky identification, macroeconomic variables do not simultaneously react to the policy variables; therefore, the macro variables will be ordered first [30]. In addition, due to the policy lags, the simultaneous reaction from the macroeconomic environment to policy variables is allowed through the ordering of the policy variables at last. This is in accordance with the Block Recursive approach proposed by [31].

5 $$ {Z}_t\left[ GDP{H}_t, INF{D}_t,E{X}_t, TE{P}_t, VOSO{L}_t,H{E}_t,M{1}_t,{R}_t\right] $$

Thus, as shown in equation [5], the first 3 variables are gross domestic product rate for healthcare and medicine sector (GDPH), inflation in the pharmaceutical sector (INFD), and free-market currency exchange rate (EX). The variable of interest (TEP) and the collection period of quests for pharmaceutical companies (VOSOL) are ordered 4th and 5th as these variables are fast-moving compared to policy variables but slow-moving compared to macro variables. The block policy variables include healthcare cost (HE), money volume (M1), and interest rates. The government mostly pays healthcare costs; therefore, it can be regarded as the fiscal policy variable. Due to the presence of fiscal dominance in the Iranian economy [32], money volume and interest rate are considered as monetary policy variables that are ordered after fiscal variables [33]. Equation [6] shows the relation between reduced-form residuals and structural shocks of the system, with A^− 1^ in a lower-triangular shape.

6 $$ \left[\begin{array}{c}{\mathrm{e}}_{\left(\mathrm{GDPH}\right)}\\ {}{\mathrm{e}}_{\left(\mathrm{INFD}\right)}\\ {}\begin{array}{c}{\mathrm{e}}_{\left(\mathrm{EX}\right)}\\ {}{\mathrm{e}}_{\left(\mathrm{TEP}\right)}\\ {}{\mathrm{e}}_{\left(\mathrm{VOSOL}\right)}\\ {}{\mathrm{e}}_{\left(\mathrm{HE}\right)}\\ {}{\mathrm{e}}_{\left(\mathrm{M}1\right)}\\ {}{\mathrm{e}}_{\left(\mathrm{R}\right)}\end{array}\end{array}\right]={\left[\begin{array}{cccccccc}{\mathrm{A}}_{11}& 0& 0& 0& 0& 0& 0& 0\\ {}{\mathrm{A}}_{21}& {\mathrm{A}}_{22}& 0& 0& 0& 0& 0& 0\\ {}{\mathrm{A}}_{31}& {\mathrm{A}}_{32}& {\mathrm{A}}_{33}& 0& 0& 0& 0& 0\\ {}{\mathrm{A}}_{41}& {\mathrm{A}}_{42}& {\mathrm{A}}_{43}& {\mathrm{A}}_{44}& 0& 0& 0& 0\\ {}{\mathrm{A}}_{51}& {\mathrm{A}}_{52}& {\mathrm{A}}_{53}& {\mathrm{A}}_{54}& {\mathrm{A}}_{55}& 0& 0& 0\\ {}{\mathrm{A}}_{61}& {\mathrm{A}}_{62}& {\mathrm{A}}_{63}& {\mathrm{A}}_{64}& {\mathrm{A}}_{65}& {\mathrm{A}}_{66}& 0& 0\\ {}{\mathrm{A}}_{71}& {\mathrm{A}}_{72}& {\mathrm{A}}_{73}& {\mathrm{A}}_{74}& {\mathrm{A}}_{75}& {\mathrm{A}}_{76}& {\mathrm{A}}_{77}& 0\\ {}{\mathrm{A}}_{81}& {\mathrm{A}}_{82}& {\mathrm{A}}_{83}& {\mathrm{A}}_{84}& {\mathrm{A}}_{85}& {\mathrm{A}}_{86}& {\mathrm{A}}_{87}& {\mathrm{A}}_{88}\end{array}\right]}^{-1}\left[\begin{array}{c}{\upvarepsilon}_{\left(\mathrm{GDPH}\right)}\\ {}{\upvarepsilon}_{\left(\mathrm{INFD}\right)}\\ {}\begin{array}{c}{\upvarepsilon}_{\left(\mathrm{EX}\right)}\\ {}{\upvarepsilon}_{\left(\mathrm{TEP}\right)}\\ {}{\upvarepsilon}_{\left(\mathrm{VOSOL}\right)}\\ {}{\upvarepsilon}_{\left(\mathrm{HE}\right)}\\ {}{\upvarepsilon}_{\left(\mathrm{M}1\right)}\\ {}{\upvarepsilon}_{\left(\mathrm{R}\right)}\end{array}\end{array}\right] $$

In this array, ε_(GDPH)_ can be interpreted as the aggregate supply shock in the healthcare sector, ε_(INFD)_ as the sectoral demand or cost-push shock, ε_(EX)_ as the exchange rate or the embargo shock, ε_(TEP)_ as the sectoral performance shock, ε_(VOSOL)_ as the collection period shock, ε_(HE)_ as the sectoral fiscal policy shock, and ε_(M1)_ and ε_(R)_ as the monetary policy shock.

Dummy variables of parliament election (MAJ_EL), presidential election (PR_EL), and health transformation program (HTP) are exogenous variables. The main instrument to analyze the effect of such variables is the historical decomposition of TEP. Historical decomposition answers the question of what proportion of the deviation of TEP from its unconditional mean is due to each structural shock. Using the Wald decomposition [34] and some backward substitutions, the variable Z_t_ in equation [3] can be modeled as a function of its initial values (Z_0_) plus all the structural shocks of the model as equation (7) [35]:

7 $$ {Z}_t={B}^t{Z}_0+\sum \limits_{k=1}^t{B}^{t-k}{\varepsilon}_k $$

## Results

**Fig. 1:**
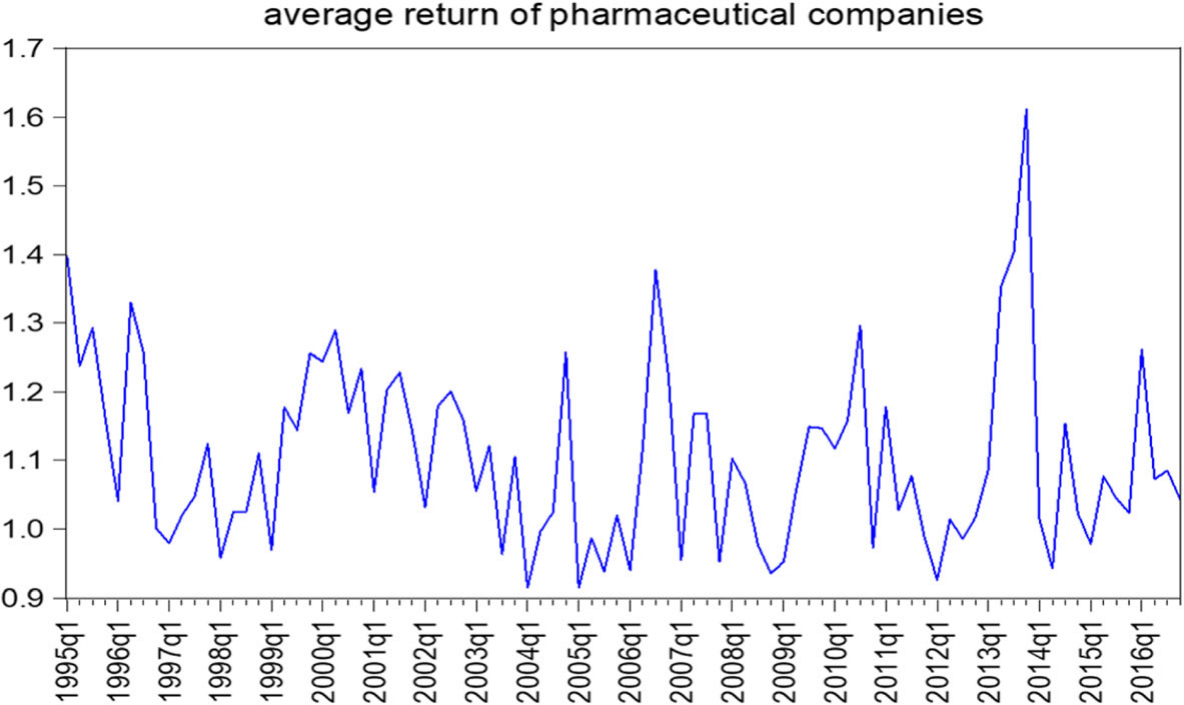
The average return of pharmaceutical companies

Currently, the pharmaceutical industry makes up 2.5% of the total value of the stock market and ranks 11th among all industries. In recent years, there has been an increase in pharmaceutical prices several times. The first price change began in 2010 and, especially in 2013, there was an increasing trend of stock prices in the pharmaceutical industry. Eventually, in 2015, a significant change in prices occurred. Figure 1 illustrates the average return of pharmaceutical companies and Table 1 compares the descriptive statistic of the research variable.

**Table 1:** Descriptive Statistics of research variables

	VOSOL	TEP	R	M1	INFD	HE	GDPH	EX
Mean	171.9107	36,821.97	0.151369	434,108.4	15.50000	5.444783	55,741.67	12,687.55
Median	186.1250	8892.546	0.140000	267,854.4	14.70000	5.502930	26,958.65	9062.950
Maximum	286.5000	228,917.8	0.200000	1,367,000.	47.40000	6.983834	217,251.7	35,791.50
Minimum	95.00000	139.8059	0.130000	30,619.90	5.400000	3.697059	1482.739	3739.200
Std. Dev.	49.91076	61,059.72	0.021421	396,293.4	7.410183	0.980374	65,360.60	9597.445
Skewness	0.104258	2.104801	1.059453	0.832028	2.066209	0.080206	1.244331	1.530400
Kurtosis	1.897090	6.149930	2.980509	2.341165	8.456747	2.002131	3.213147	3.764724
Jarque-Bera	4.409610	96.74984	15.71550	11.21100	163.9854	3.575165	21.83606	34.83654
Probability	0.110272	0.000000	0.000387	0.003678	0.000000	0.167364	0.000018	0.000000
Sum	14,440.50	3,093,046.	12.71500	36,465,105	1302.000	457.3618	4,682,300.	1,065,755.
Sum Sq. Dev.	206,760.0	3.09E+ 11	0.038086	1.30E+ 13	4557.598	79.77412	3.55E+ 11	7.65E+ 09
Observations	84	84	84	84	84	84	84	84

In this part of the study, to avoid delusive regression in the model, the existence of unit root in research variables is examined. The test results in Table 2 show that in case of collection period of quests growth rate (VOSOL) and interest rate (R), in both ADF and PP tests, due to lower values of measures rather than critical values in significance level of 95%, these variables have a unit root at the surface. In addition, H_0_ indicates that the existence of unit root is not denied, and these variables become stationary with a single differentiation. Furthermore, such variables are accumulated from the order of 1 or I (1). Also, in the ADF test, the rate of healthcare cost growth is non-stationary but becomes stationary with a single differentiation. Other variables are stationary; i.e., I (0).

**Table 2:** Unit root test for research variables

Result	1st difference			Level			Variables
	Coefficient	Critical values at 5%	Prob	Coefficient	Critical values at 5%	Prob	
ADF Test							
I (0)	–	–	–	−6.48	−2.85	0.0000	TEP
I (0)	–	–	–	−4.96	− 2.89	0.0001	GDPH
I (0)	–	–	–	− 4.46	−2.89	0.0005	INFD
I (1)	−11.54	−2.89	0.0001	−0.24	−2.89	0.9277	HE
I (0)				−3.49	−2.89	0.01	M1
I (1)	−7.10	−2.89	0.0000	−2.51	− 2.89	0.1157	r
I (0)				−6.11	−2.89	0.0000	EX
I (1)	−3.79	−2.90	0.0045	−1.48	−2.89	0.5388	vosol
Philips-Perron							
I (0)	–	–	–	6.4841-	2.8951-	0.0000	TEP
I (0)	–	–	–	5.0343	2.8972-	0.0001	GDPH
I (0)	–	–	–	2.7177-	2.5853-	0.0753	INFD
I (0)	–	–	–	6.7554-	2.8959-	0.0000	HE
I (0)	–	–	–	17.49-	2.8951-	0.0001	M1
I (1)	2.6446-	2.5826-	0.0883	1.5071-	2.8963-	0.5253	r
I (0)				6.1142-	2.8967-	0.0000	EX
I (1)	4.6572–0	2.8967-	0.0002	1.4804-	2.8959-	−0.5388	vosol

To determine the proper lag length in convergence test, Likelihood Ratio (LR), (FPE), Akaic (AIC), Schwartz (SC), and Hannan-Queen (HQ) measures were used. Table 3 presents the results for choosing the lag length. The results indicate that the optimum lag length is equal to 2. After determining the optimum lag length, the next step is to perform a convergence test.

**Table 3:** Determining the optimum lag

Lag	LogL	LR	FPE	AIC	SC	HQ
0	154.0983	NA	122.39 e -	4.058287-	3.805324-	3.957581-
1	497.1921	600.4140	1.04e-15	11.81089	9.534224-	10.90454-
2	647.9562	230.3341	9.90e-17*	14.22101-	-^*^9.920637	-^*^12.50902
3	707.6810	77.97405	1.32e-16	14.10225-	7.778178-	11.58462-
4	789.2472	88.36335	1.15e-16	^*^14.59020-	6.242424-	11.26693-

* show the optimum lag that every test recommended that

In the next step, we intend to assess the existence of a long-term relationship between variables. For this purpose, Johanson’s convergence test for time series data is used. In this phase, the number of accumulation vectors between model variables is determined using values of matrix effect and maximum specific values (Table 4). Regarding these tests, the existence of r accumulation vectors (i.e., H_0_ assumption) is accepted when the quantity of this statistic is lower than the critical value provided by Johanson and Jucilious. Hence, according to the above tables and considering both statistical values, the effect and maximum specific values of two accumulation vectors exist between model variables.

**Table 4:** Convergence test

Null hypothesis	Alternative hypothesis	Coefficient	Critical values 95%	Prob
Trace Test				
r = 0	1 = r	264.9716	159.5297	**0.0000**
r≤1	2 = r	168.5711	125.6154	**0.0000**
r≤2	3 = r	95.74869	95.75366	**0.0500**
r≤4	4 = r	62.09743	69.81889	**0.1767**
r ≤ 4	5 = r	40.41922	47.85613	**0.2079**
***λ***_***max***_ Test				
r = 0	r ≥ 1	96.40047	52.36261	**0.0000**
r ≤ 1	r ≥ 2	72.82243	46.23142	**0.0000**
r ≤ 2	r ≥ 3	33.65125	40.07757	**0.2211**
r ≤ 3	r ≥ 4	21.67821	33.87687	**0.6325**
r ≤ 4	**r ≥ 5**	**3.525272**	**3.841466**	**0.0604**

**Fig. 2:**
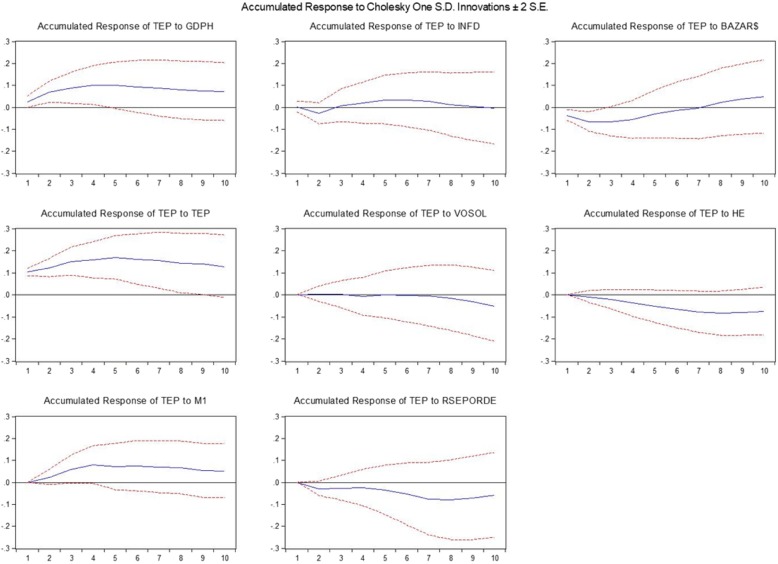
Responses of TEP to endogenous economic shocks

Figure 2 presents the IRFS of the VAR models. To check the existing dynamicity between pattern variables, response functions are used. These functions are the responses coming from an internal variable of the system toward the shock caused by errors. These functions determine the effect of a unit shock as much as a standard deviation over current and future values of the endogenous variable. On this basis, the effect of a unit random shock of research variables on the stock return of pharmaceutical companies is examined.

When a macro positive shock occurs to GDPH, inflation, and currency rate, it increases the stock return in the long run. The positive effect of GDPH on the stock returns is sustained over time. Increasing production causes increasing the income flow of the companies and their profitability, leading to the positive reaction of the stock market index in comparison to production. In contrast, a positive shock to inflation and exchange rates initially reduce stock returns. The currency rate effect also complies with theoretical fundamentals. In this regard, by increasing the currency rate in the short term, the costs associated with procuring raw materials increase and the profitability of the companies fall.

When a financial sector shock (e.g., VOSOL) occurs, the stock return of these companies declines. This effect increases over time and becomes permanent.

In the case of policy-making shocks such as health expenditure (fiscal shock) or interest rate and money volume (monetary shock), the result is different. The shock incurred to healthcare costs and interest rates leads to decrease stock return of the companies. This also complies with reality, because bank deposits are considered as a replacement asset for stocks and an increase in their interest rate persuades investors to make a deposit in banks which reduces demands for stocks. Moreover, the bank interest rate as a portion of the discount rate also explains the negative reaction of the stock index to the bank interest rate. A positive shock on the money volume leads to an increase in the stock return that is consistent with the theory.

By variance decomposition of prediction error, we can find out the extent to which the changes in a sequence are affected by the disturbance components of the sequence itself, and how much it is affected by the disturbance components of other variables inside the system. According to the results of variance decomposition (Table 5), in the short-term (about 2 periods), a high proportion of stock market return is described by the variable itself. However, other variables of the model in this period do not provide significant description of stock fluctuations and their descriptive nature is negligible. Such results show the relative and short exogenous nature of such variables in relation to model variables. However, in long-term and after 10 periods, descriptive power of market currency rate, money volume, pharmaceutical sector inflation, bank interest rate, GDP of healthcare sector, healthcare costs, and collection period of quests from return index of stock market is 10.99, 9.18, 7.56, 6.87, 5.05, 4.26, and 2.72%, respectively.

**Table 5:** Variance decomposition

HE	VOSOL	R	M1	INFD	GDPH	EX	TEP	S.E.	Period
0.000000	0.000000	0.000000	0.000000	0.000000	0.000000	0.000000	100.0000	0.111224	**1**
(0.00000)	(0.00000)	(0.00000)	(0.00000)	(0.00000)	(0.00000)	(0.00000)	(0.00000)		
0.643716	0.516556	3.645996	4.275571	3.045998	6.745288	6.498856	74.62802	0.135257	**2**
(1.58969)	(2.47750)	(4.14222)	(4.38718)	(4.44909)	(5.81670)	(5.83215)	(10.0833)		
1.885852	0.552555	3.125592	8.092319	5.987067	6.270742	7.027835	67.05804	0.147539	**3**
(2.29192)	(3.23537)	(3.69456)	(5.87893)	(5.47160)	(5.30892)	(5.48121)	(9.82294)		
3.113979	0.882011	3.254085	9.538053	6.176203	6.241883	7.927058	62.86673	0.152701	**4**
(2.96393)	(3.82667)	(4.22017)	(6.31393)	(5.72438)	(5.21573)	(5.63885)	(9.78998)		
3.665863	0.871941	3.813322	9.110137	5.895478	5.909447	11.21041	59.52340	0.156943	**5**
(3.07758)	(4.05572)	(5.02856)	(5.73867)	(5.56695)	(5.02425)	(6.75392)	(9.59936)		
4.106707	1.026344	4.940055	8.804775	5.910681	5.702113	11.58808	57.92125	0.159773	**6**
(3.20950)	(4.29927)	(6.30071)	(5.47682)	(5.55962)	(5.04474)	(6.64298)	(9.35905)		
4.479130	1.531116	6.063675	8.625048	5.797920	5.590064	11.31098	56.60207	0.162475	**7**
(3.27950)	(4.62186)	(7.17887)	(5.28394)	(5.59362)	(5.07553)	(6.49288)	(9.31475)		
4.354149	1.801515	5.850278	8.378294	7.041856	5.346722	11.37644	55.85075	0.166133	**8**
(3.25200)	(4.89775)	(7.53143)	(5.21896)	(5.93316)	(5.02693)	(6.50874)	(9.28173)		
4.307578	2.112254	6.193077	9.156914	7.479709	5.158353	11.29346	54.29866	0.169156	**9**
(3.15717)	(5.15227)	(7.45971)	(5.36344)	(6.27615)	(5.00786)	(6.60511)	(9.20960)		
4.266167	2.729970	6.877257	9.182257	7.560340	5.051597	10.99235	53.34006	0.171478	**10**
(3.12107)	(5.34217)	(7.70037)	(5.34699)	(6.44625)	(4.98286)	(6.52365)	(9.24829)		

**Fig. 3:**
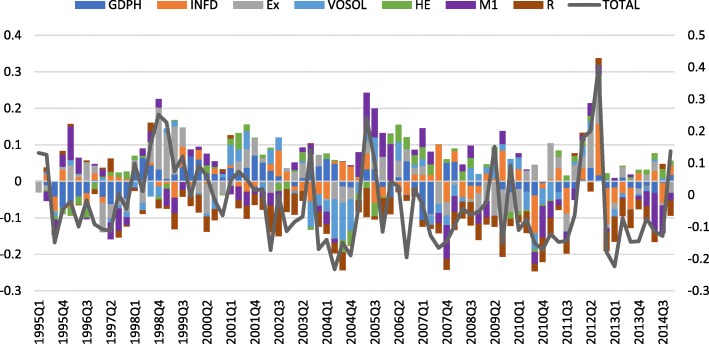
Historical decomposition of TEP

As the effects of exogenous non-economic variables cannot be analyzed through IRF, historical decomposition of TEP is used to investigate the effects of such variables. According to Fig. 3, among the dummy variables, the dummy of the government elections during the years 2005 to 2007 has led to an increase in the share of monetary shocks in stock returns fluctuations. Such an increase is attributed to the expansionist policies of the new government. The parliamentary election dummy variable does not show a significant impact on stock returns.

Among the non-economic factors, the health transformation program has had a significant effect on fluctuations in stock returns and increased the collection period of quests for pharmaceutical companies during the period of 2014–2015. As a result, fluctuations in returns increased due to the impact of financial shocks.

Outside of the mentioned periods, the exchange rate shocks, inflation shock, money supply shock, and supply shock of the health sector have most to explain the stock return volatility. For example, during the period of 2012–2013, with the increase in economic sanctions and the exchange rate swing, stock returns declined initially but gradually increased thereafter.

## Discussion

This article examines the effect of major economic and non-economic variables over the stock return of pharmaceutical companies in the Tehran Securities Exchange market using 1995–2016 quarterly data. Pharmaceutical companies in the stock market are affected by various elements including two groups: internal and external elements. The present study is aimed at examining the way such variables affect the stock price index of pharmaceutical companies using a VAR model.

According to Chue and Cook (2008), the relationship between stock return and currency rate in developing markets is different according to various periods [36]. The obtained results can be perceived as by increasing the currency rate in the short-term. In this regard, costs associated with procuring raw materials increase and companies’ profitability decreases. Due to the over-importing of pharmaceutical companies, the risk of fluctuation in the currency rate has a significant impact on these companies such that over 50% of raw materials for pharmaceutical companies are imported from abroad. Any changes in the currency rate used in transactions lead to a change in the final price of the companies. This complies with the results of Boswilhem et al. (2011), Karimzadeh (2006), and Qalmeq (2005) [37–39].

An increase in economic development is associated with an increase in the income flow of companies and their profitability, which finally causes a positive reaction of the stock index compared to production. Obviously, the more economic stability increases in a country, the tendency to invest among people will be higher such that the companies will act more confidently in implementing their projects. Such factors cause investment in the securities exchange market to change following the general economic status of the country. The level of actual activities as another effective element on stock price, according to Gordon’s equation, is directly related to stock price and is used in this regard [17].

The positive shock incurred in money volume leads to an increase in the stock outcome of pharmaceutical companies. Thornback reports similar results, stating that expansionary monetary policy leads to an increase in stock return [40]. Also, Patelis’s findings show that contractionary monetary shocks lead to lower tock return [41]. Based on common theories, an increase in money volume leads to a decrease in the interest rate and leads to an increase in stock price through a decrease in the discount rate. However, this equation may not be the same in the long term because in this way money volume leads to an increase in the level of prices and enhancing inflation [25].

Bank deposits are replacing equity for stock. In this context, an increase in their interest rate encourages investors to make a deposit in banks, leading to a decrease in demands for the stock. Also, the bank interest rate is considered as a part of a discount rate, which justifies the negative reaction of the stock index in relation to the bank interest rate. This result is compatible with Boswilhem et al. (2011) and Karimzadeh (2006). According to Assefa (2017), changes in interest rate have a negative effect on stock return in both developed and developing countries. They indicate that this negative relationship is intuitive due to the following reasons. Evaluation model of free cash flow estimates fundamental value using a discount rate; when this rate increases, the current value of expected flow decreases, leading to a decrease in stock price. In comparison, it increases the discount rate lower than the stock return. As an additional channel, an expansionary monetary policy increases the money supply and thus the effect of wealth is transferred to the stock market which provides a positive outcome [16]. Other scholars such as Fama and Schwert (1977) and Flannery and James (1984) show the negative effects of interest rate on stock return, particularly according to the inflation component of the inflation rate [42, 43].

Examining financial statements and balance sheets of pharmaceutical companies shows the continuum of an incremental trend in the collection period of quests for such companies. The average collection period of quests for 22 pharmaceutical companies in 2015 was 252 days. Meanwhile, between March to Jun 2016, this period reached 299 days and witnessed an 18% increase, i.e. 46 days. It seems that a lack of resources for healthcare insurance is the most important factor in increasing the collection period of quests for pharmaceutical companies. Moreover, it should be noted that the public sector, especially medical sciences universities, have a large amount of undue payments and the process of settlement in such centers or flow cycle of payable accounts is so long in such sectors. Accordingly, this issue causes monetary pressures on pharmaceutical companies, particularly distributors who cooperate with them.

The health transformation program influences the stock index of pharmaceutical companies because this plan increases the collection period of quests for pharmaceutical companies, leading to a decrease in stock return.

The results of variance decomposition show that in the long-run, market currency rate, money volume, pharmaceutical sector inflation, interest rate, medical sector GDP, healthcare costs, and collection period of quests can better describe the power of stock market return index, in the order of their appearance.

## Conclusion

After the petroleum and petrochemical industries, the pharmaceutical industry is the second most profitable in the world. This technology is considered a high-tech knowledge-based sector of the economy. Therefore, for R&D activities regarding this sector, it is necessary to allocate large investments.

This study examines the effect of economic and non-economic factors on the stock return of pharmaceutical companies in the securities market of Iran. For this purpose, an autoregressive model (VAR) with Cholesky identification, data of economic and non-economic variables, and the return of pharmaceutical companies in the Tehran Securities Exchange market from 1995 to 2016 were used.

To examine the stationarity of variables, Dicky-Fuller and Philips-Perron tests were used and found that there is a combination of stationary and non-stationary variables in this regard. Then, to examine proper lag length in convergence test, Likelihood Ratio (LR), (FPE), Akaic (AIC), Schwartz (SC), and Hannan-Queen (HQ) measures were used. The results show that the optimum lag length is equal to 2. Then, using statistics of the test, the effects of matrix and maximum specific values were identified. According to both statistics, there are two accumulation vectors between model variables.

The results of the present show that positive shock incurred at the currency rate, bank interest rate, collection period of quests, and healthcare costs lead to a decrease in stock return of pharmaceutical companies. On the other hand, positive shock incurred atGDP and money volume lead to an increase in the return of pharmaceutical companies.

The results show the different reactions of variables in a certain period. Our analysis has some implications for policymakers. For example, the relationship between the exchange currency rate and stock return is not stable throughout the time but the currency rate has a significant impact on the fluctuations of the stock price index of pharmaceutical companies. Therefore, policymakers are suggested putting the supply of currency resources for pharmaceutical companies on the agenda or float the exchange rate, which prevents currency mutations and its negative short-term impact on the industry’s returns.

Also, before implementing large-scale financial policy initiatives, such as the Health Transformation Program, it is highly recommended full financing the required resources in the first place before implementing such plans. The results show that in the case of Iran, implementation of this plan, despite the benefits for the low-income population, resulted in an increase in the company’s collection period of quests, which has had a negative effect on the volatility of the industry’s returns.

Considering the liquidity shortages as a major problem in Iran and insufficient pharmaceutical R & D field due to credit scarcity, quantitative easing of monetary policy, like those of the year 2005–2007, can be effective in increasing the efficiency of the industry. In fact, the credit channels of monetary policy such as the Balance Sheet channel and Bank Lending channel are vital in affecting the industry. In this regard, higher interest rates lead to a decrease in the net worth of borrowers (due to higher debt burden) and a decrease in the value of the assets of lenders. As a result, the demand for and supply of loans can decreases. This can lead to further changes in asset prices through the so-called Financial Accelerator mechanism [44]. A decline in the net worth of firms reduces the value of the collateral that firms can use to borrow, resulting in tighter credit conditions. As a result, lower investment and economic activity may occur, which further depresses firms’ profitability and net worth. Moreover, it may lead to the further tightening of financial conditions by lenders, thereby amplifying the contractionary impact of the initial interest rate increase. As a secondary effect, monetary policy tightening can, therefore, raise credit and financial stability risks. Besides, monetary policy may affect the supply of loanable funds available to banks and thus the amount of loans banks can create. Banks play a special role in the economies like Iran in which few substitutes exist for bank loans. Thus, a tighter supply of bank loans or tighter credit conditions would again weigh on spending and investment.

Given the lack of uniformity of results in short-term and long-run effects for some of the model variables, it is suggested considering both current and future situations. Both short-term and long-term effects should be considered in policymaking such that long-term effects on society due to a positive effect in the short run would be avoided.

Moreover, our results are related to stakeholders in the stock market: perceiving the relationship between affective variables on stock price is necessary for the purpose of portfolio management and risk management.

Based on the findings of this study, the following actions are recommended to be done by the policymaker;

1. Exercising a managed-floating exchange rate system to prevent massive fluctuations in the industry;
2. Securing the financial resources for the implementation of large-scale fiscal plans such as the Health Transformation Program to decrease the collection period of quests in the industry; and
3. Monetary easing policy to dampen the credit scarcity and easing the R&D activities in the industry, which finally can lead to high-quality or cheaper products.

Our study is subject to some limitations. First, the results would be more robust, providing a more extended data horizon. Second, modeling the dynamics of sanctions in Iran is somehow not practicable.

Due to the relationship between different economic sectors, it is appropriate a topic for future research to examine the research question via a Panel Data model to investigate the effects of macroeconomic variables on a micro-scale. Another proposed topic is to investigate the effect of sanctions or trade limitations via a general equilibrium model such as DSGE. The further model can capture the fundamental issues affecting the industry.

## Data Availability

The datasets used and/or analyzed during the current study are available in public access. https://www.cbi.ir/section/1378.aspx https://www.amar.org.ir/%D8%AF%D8%A7%D8%AF%D9%87%D9%87%D8%A7-%D9%88-%D8%A7%D8%B7%D9%84%D8%A7%D8%B9%D8%A7%D8%AA-%D8%A2%D9%85%D8%A7%D8%B1%DB%8C https://codal.ir/

## Abbreviation

SVAR: Structural Vector Auto-Regressive
